# Particle slip velocity influences inertial focusing of particles in curved microchannels

**DOI:** 10.1038/s41598-018-30171-9

**Published:** 2018-08-07

**Authors:** Saurabh Deshpande, Phanindra Tallapragada

**Affiliations:** Department of Mechanical Engineering, Clemson University, South Carolina, USA

## Abstract

Size based separation and identification of particles in microfluidics through purely hydrodynamic means has gained significant interest due to a number of possible biomedical applications. Curved micro-channels, particularly spiral micro-channels with rectangular cross-section and the dynamics of particles in such channels have been extensively researched to achieve size based separation of particles. In this paper we present evidence that sheds new light on the dynamics of particles in such curved channels; that a unique particle slip velocity is associated with the focusing positions in the cross sections, which leads to a balance of forces. Our experiments therefore imply that the forces acting on the particle lead to convergence to an attractor in both the physical space (the cross section of the channel) and the slip velocity space.

## Introduction

The motion of spherical particles in a channel leads to preferential migration or focusing in a small region of the cross section^1^. This phenomenon of inertial focusing has received much attention in the last decade in the context of microfluidics due to their potential biomedical applications such as for high through put identification or separation of cells. Curved microchannels, in particular spiral microchannels with a rectangular or square cross section have been extensively researched^2–6^ for this purpose. Particles are known to focus in a region of the cross section where the lift force and Dean drag balance each other and properly selecting the geometry of the channel and flow rates achieves controlled focusing of particles.

When fluid flows through a channel whose centerline is curved, a secondary flow arises within the cross section of the channel. The curvature ratio is defined as $\delta =\frac{{d}_{h}}{{d}_{c}}$ where *d*_*h*_ is the hydraulic diameter of the channel and the *d*_*c*_ is the diameter of the channel centerline. The relevant non dimensional parameters of the flow are the channel Reynolds number $Re=\frac{U{d}_{h}}{\nu }$ and the Dean number, $De=Re\sqrt{\delta }$ which combines the influence of the Reynolds number and the channel curvature. Here *U* is the average axial velocity of the fluid and *v* its kinematic viscosity. The secondary flow takes the form of two vortex cells, the so called Dean vortices, when the channel Reynolds number and the Dean number are small^7^. When small spherical particles of diameter *d*_*p*_ are injected into a curved pipe they do not necessarily circulate in the cross section with the same velocity as the secondary flow. This difference in the cross sectional relative velocity causes the Dean vortices to exert a drag force on them called the Dean drag force, *F*_*D*_, in a direction perpendicular to the centerline. A second force on the particle in a direction perpendicular to the axial line is the lift force. The lift force is cause due to the curvature of the flow. Particles migrate to a positions where the lift force and Dean drag balance each other. These new equilibrium positions depend on the particle confinement ratio, $\lambda =\frac{{d}_{p}}{{d}_{h}}$, the channel aspect ratio, the channel Reynolds number and the Dean number^2^.

Many theoretical studies, see for example^8–10^, in the last few decades have established that the lift force on a particle in a shear flow or a Poiseuille flow depends nearly linearly on the slip velocity of the particle in the axial direction. Particle slip velocity is defined as the relative axial velocity of the particle with respect to the undisturbed fluid axial velocity. Within the inertial microfluidics literature scaled relationships of the lift force have been developed that depend only on the position of the particle in the channel cross section, see for instance^2,3^. These scaling relationships ignore the factor of particle axial slip velocity entirely. In this paper we present evidence that the phenomenon of particle focusing is also influenced by the particle axial slip velocity. Unrelated to inertial particle focusing, it is known that neutrally buoyant particles lag the flow in a Poiseuille flow^11^. This is however the first work where we provide experimental evidence that particles have an axial slip velocity in a curved spiral channel. We show that distinct particle axial slip velocities are associated with particle spatial focusing positions in a spiral channel’s cross section, i.e. particle focusing phenomenon is due to a convergence of particle dynamics spatially as well as in slip velocity.

## Background on Particle Slip Velocity and Focusing

Taking a dynamical systems point of view, several papers^12–16^, have predicted the clustering and size based segregation of particles in some canonical two and three dimensional fluid flows. The calculations in these papers are based on a simplification of the Maxey-Riley equation^17^, that governs the motion of a small spherical neutrally buoyant particle,

1 $$\frac{d}{dt}({\bf{v}}-{\bf{u}})=-\,[({\bf{v}}-{\bf{u}})\cdot \nabla ]{\bf{u}}-\,\frac{2}{3}S{t}^{-1}({\bf{v}}-{\bf{u}})$$

where **v** is the velocity of the particle and **u** is the undisturbed velocity of the fluid at the same location, *St* is the particle Stokes number. The particle Stokes number is a measure of the inertia of the particle. It depends on the particle diameter *d*_*p*_ and the fluid Reynolds number *Re* through the equation $St=\frac{2}{9}Re\frac{{d}_{p}^{2}}{{L}^{2}}$, where *L* is a length scale. The length scale relevant to particle focusing in a microchannel is the hydraulic diameter, *d*_*h*_, of the channel. So the particle Stokes number is $St=\frac{2}{9}Re{\lambda }^{2}$. The position of the inertial particle **r** = (*x*, *y*, *z*) is determined by the equation

2 $$\frac{d{\bf{r}}}{dt}={\bf{w}}+{\bf{u}}$$

where **w** = **v** − **u** is the relative velocity (or slip velocity) of the particle. Equation (1) describes how the velocity of a particle, **v**, evolves in a fluid flow with velocity **u**. Particles at a position **r** could have a velocity that is different from a hypothetical fluid particle located at the same location. The slip velocity **w** = **v** − **u** is the difference between the particle velocity and the undisturbed velocity of the fluid at the particle location if the particle were absent. It should be noted that the fluid truly never slips along the surface of a particle in this Reynolds number regime. It has been shown that despite transients and chaotic excursions the particle’s relative velocity eventually decays to zero in steady flows or converge to a slow manifold in unsteady flows and that particles cluster in certain regions of the flow^12,14,18^.

Equation (1) ignores the lift force on the particle arising due to inertia and walls or boundaries. If the axial velocity of the particles leads or lags the flow, **w** ≠ 0, then the particles also experience a lift force, *F*_*L*_ in the transverse direction. The lift force on a neutrally buoyant spherical particle has several contributing factors. Saffman^8^, found that a particle moving with a slip velocity *W* in a linear shear flow experienced a lift force that was proportional to the shear gradient *γ* and the slip velocity. The direction of the lift force is in the direction where the fluid’s relative velocity is greater. Several analytical refinements extending Saffman’s formula for the lift force on a spherical particle in Poiseuille flow, show that the lift force to increases with the slip velocity of particle while still being nonzero at zero slip velocity^9,10,19,20^.

Computational results for instance those in^11,19^ which include strongly nonlinear effects show that neutrally buoyant particles lag the undisturbed flow and experience a hydrodynamic lift force towards the channel walls. The dynamics of particles in curved channels can be more complex. When fluid flows through a channel whose centerline is curved, a secondary flow arises within the cross section of the channel. When small spherical particles are injected into a curved pipe they experience a Dean drag force, *F*_*D*_, in a direction perpendicular to the centerline of the channel due to the Dean vortices. If the Dean drag force, the shear gradient induced lift force and the wall induced repulsion sum to zero at some point in the channel cross section, an equilibrium position for these particles is created. If such an equilibrium position is stable, particles will migrate to it, or focus at this position. We show through our experiments that a necessary condition for such an equilibrium position could be that the particle slip velocity be nonzero.

## Experimental Procedure

**Figure 1:**
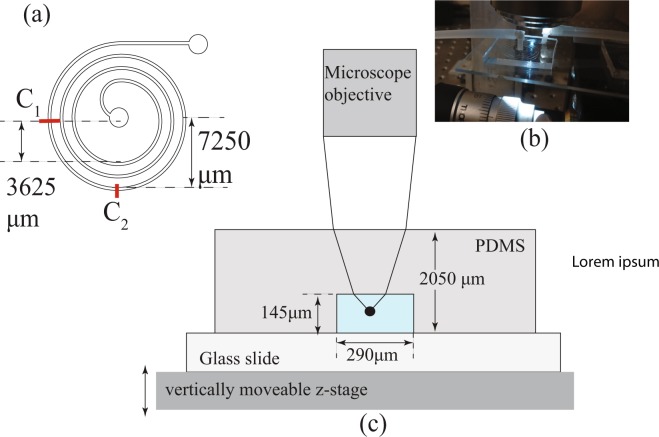
Experimental setup. (**a**) Top view of the spiral microchannel with the inlet at the center, (**b**) a sample device, (**c**) schematic of the cross section of the device and refracting light rays that travel to the objective lens of the microscope The geometry of the device in (c) is not to scale. The particle images in the channel are recorded at section *C*_1_ on the last spiral arm in (a).

A schematic of the top view of the microchannel device is shown in Fig. 1(a). The curvature ratio varies from 0.04 to 0.02 while the channel is traversed from the innermost spiral arm to the outermost arm. The device is made of Polydimethylsiloxane (PDMS) via a standard soft lithography process. Water containing rigid spherical micro particles of diameter 24 *μ*m (*λ* = 0.1214) made of polyethelene with density 1 g/cm^3^ at a volume fraction of 0.03% was pumped through this microchannel at several constant flow rates until particle focusing was achieved. The small confinement ratio of the particles together with the small particle volume fraction ensures that the channel is not blocked by a particle or the velocity of the fluid in the channel is not significantly altered far away from the particle. The device is placed on a 3-axis flexure stage with differential micrometers that allow vertical motions of the stage with an accuracy of 1 *μ*m. Figure 1(b) shows a picture of this physical setup.

We identified the screw gage reading of the differential micrometer for the baseline case of the channel bottom being at the focal plane of the objective. We did this by having markings on the glass slide before the PDMS device was bonded to the slide. Thus the zero height of the z-stage is calibrated. The change in height of the focal plane of the objective, Δ*z*_*f*_ as the z-stage moves by a distance of Δ*z*_*s*_ are related by Snell’s Law. A light ray would travel through three media: air, PDMS and water, as shown in Fig. 1(c). The refractive index of air is taken as *μ*_1_ = 1 since the experiments were performed at a nearly constant temperature of 22 °C. The refractive index of PDMS is taken as *μ*_2_ = 1.4 (from data sheet of manufacturer, Dow Corning) and that of water is taken as *μ*_3_ = 1.33. The thickness of the PDMS slab above the channel is 1905 *μ*m and the numerical aperture of the objective lens is 0.6. Calculations show that for this thickness of PDMS, Δ*z*_*f*_ = 1.56Δ*z*_*s*_. We confirmed the values of the refractive index of the PDMS by fabricating several channels with different heights and calibrated the height traveled by the objective to the top of the device. We performed these measurements with and without fluid in the channel, to calibrate for the effects of the refractive index of water.

**Figure 2:**
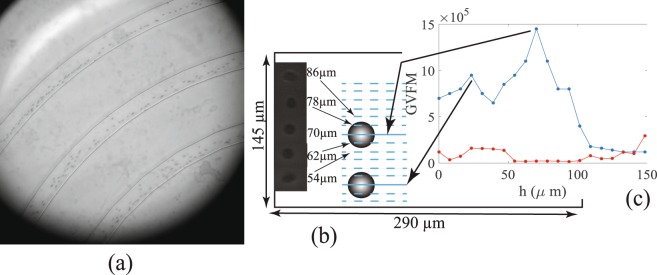
(**a**) A sample image of particles focused in the outermost spiral arm. (**b**) Sample images of particles at different heights in the vertical stack. (**c**) The right panel shows the average GVFM of images of particles at different heights by the (top) blue curve. The (bottom) red curve is average GVFM at different heights in the absence of particles. The local maxima in the top curve indicate the heights at which the particles are best focused.

Particles that are pumped into the channel with a random distribution in the cross section with a random distribution of velocities eventually attain an equilibrium position with respect to the lateral walls of the channel. According to eq. (1) the convergence of particles to this equilibrium is not necessarily exponentially fast and requires a finite time during which the particles traverse a long distance along the channel. Experimentally the necessity of a ‘focusing length’ for the transient dynamics of the particles to decay in curved channels has been well known^2,3,21,22^. This is demonstrated in Fig. 2(a) where the particle distribution is less focused in the inner spiral arm (the channel arm with the smallest arc length) and are progressively focused laterally, i.e., reach a nearly uniform distance from the inner wall as they flow into the outer spiral arms. Particles consistently attain spatial equilibrium positions in the cross section in the outer most spiral arm in Fig. 2(a) and all the subsequent measurements of particle lateral positions, and velocities are at a fixed location, *C*_1_, shown in Fig. 1(a) of the channel in the outer most spiral arm.

The heights of the particle equilibrium positions are measured from the bottom of the channel. To do this images are recorded at regular height intervals in the channel to observe how well focused the particles are in the images. The microscope objective lens (Nikon TU Plan Fluor EPI ELWD 50X) used in the height measurements has a 50*X* magnification and a depth of focus of 0.9 *μ*m, allowing one to discriminate between the quality of an image when the objective moves relative to the device by even a few micrometers. A vertical stack of images of particles in the spatial equilibrium positions is assembled as shown in Fig. 2(b). We then quantify the sharpness of the boundary of the particles and the size of the particles in the vertical stack of images to identify the height at which the particles are best focused. This is done by quantifying the variance in the pixel values which range from 0 to 255 in the image. The local variance in pixel intensity level in gray scale image is measured at each pixel using a sliding window. The method is briefly explained below. Suppose the intensity of the pixel located at (*i*, *j*) in an image of size *M* × *N* is denoted by *f*(*i*, *j*). A sliding window of size *a* × *b* pixels centered around each pixel in the image is chosen. A sub image of the original image is first selected such that the sliding window can always fit at every pixel of the sub image. The indices of the pixels of this sub image are from *M*_0_ = *a*/2, *M*_*f*_ = *M* − *a*/2, *N*_0_ = b/2 and *N*_*f*_ = *N* − *b*/2. The average value of the pixel intensity in this window centered at (*i*, *j*) is denoted by $\overline{f}(i,j)$. The local variance of the pixel in this sliding window is

3 $$LV(i,j)=\frac{1}{ab-1}\sum _{{y}_{2}}^{{y}_{1}}\,\sum _{{x}_{2}}^{{x}_{1}}\,(f(p,q)-\overline{f}(i,j))$$

where ${x}_{1}=\lceil i-a\mathrm{/2}\rceil$, ${x}_{2}=\lfloor i+a\mathrm{/2}\rfloor$, ${y}_{1}=\lceil j-b\mathrm{/2}\rceil$, ${y}_{2}=\lfloor \,j+b\mathrm{/2}\rfloor$, represent the corner pixel numbers of the sliding window and *p*, *q* are pixel indices within this sliding window. The variance of these local variances is the global variance focal measure (GVFM) of the image^23^. Thus

4 $$GVFM=\frac{1}{\alpha }\sum _{p={M}_{0}}^{{M}_{f}}\,\sum _{q={N}_{0}}^{{N}_{f}}\,(LV(p,q)-\overline{LV})$$

where $\overline{LV}$ is the average value of the local variances obtained in (3) and *α* is the total number of sliding windows. The GVFM calculations for our experiments are done through programs written in MATLAB. Images of particles in a vertical stack for which the GVFM is a local maxima have sharp well contrasted boundaries and correspond to the case where they are best focused^23^. It thus allows one to estimate with a very high degree of precision the vertical position of a particle in the channel. In Fig. 2(b) the blue graph measures GVFM of the image in a stack of images whose depth varies from the bottom wall to top wall of the channel at intervals of about 8 *μ*m. The graph has two maxima one at a height of 23 *μ*m and the other at a height of 70 *μ*m. These are the heights from the bottom of the channel where the particle images are the most well focused indicating that the particles focus at these heights. For reference the GVFM of a similar stack of images for the flow without any particles in shown in Fig. 2(b) by the red graph. This graph is nearly uniform with a low value of GVFM and no local maxima.

A Photron FastCam SA4 camera recording at rates of 9000 to 13500 frames per second at a resolution of 512 × 512 was mounted on a Nikon microscope to record the motion of the particles. The velocity of the fluid in the channels in these experiments is of the order of 0.1 m/s. Since the velocity of the particles is of the same order as that of the fluid, the frame rate of the camera is sufficient to record changes in the position of the particles on the order of a few micrometers. The velocity of a particle at a focused position is measured by tracking the pixels of the center of the particle’s image in the consecutive frames. The identification and tracking of centers of spheres is done through MATLAB programs using the image processing toolbox.

**Figure 3:**
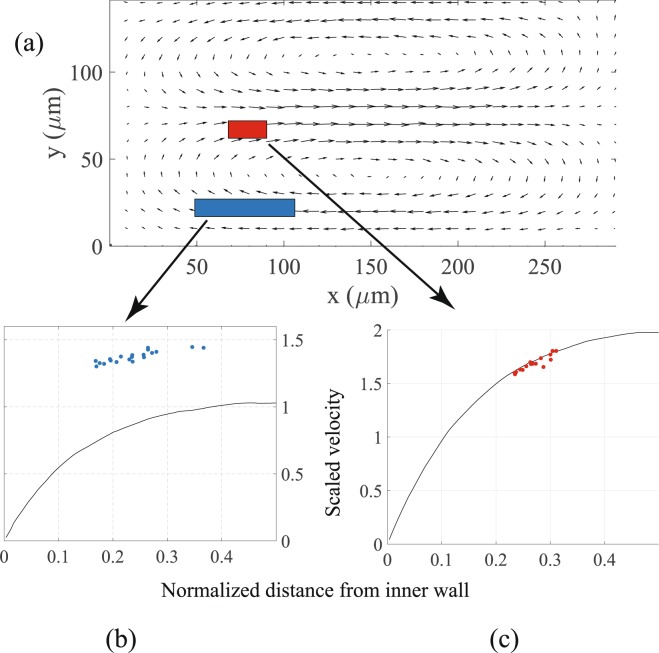
(**a**) Positions of focusing windows in the cross section with the Dean vortices. The inner wall is at *x* = 0 and the bottom of the channel is at *y* = 0. (**b**) and (**c**) The velocities of the particles in the two focusing windows, with the black curve showing the velocity of the fluid.

Particles of diameter 24 *μ*m attain tightly focused equilibrium positions at about *Re* = 12.4. Once this particle focusing is attained the focal plane of the objective is located at the height where the GVFM has a maxima. This maxima occurs at two different heights in the channel, the first is at a height of 23 *μ*m and the second is at a height of 70 *μ*m from the bottom of the channel. Figure 3(a) shows two focusing windows (a blue and a red rectangle) at these positions within the cross section to which the particles converge.

The undisturbed velocity of the fluid is computed through a numerical simulation in COMSOL Multiphysics. In the small Reynolds number regime, the flow through an axially symmetric pipe with steady boundary conditions can be accurately computed through commercial softwares such as COMSOL. In the present study, a numerical simulation in the software COMSOL was used to simulate the Newtonian fluid flow in the channel. The simulation used an iterative GMRES solver with a tolerance of 10^−4^. An adaptive mesh was used with the maximum element size being 7 *μ*m. The axial velocity of the fluid and velocity of the secondary flow were computed in COMSOL at the cross section *C*_1_ (Fig. 1(a)) at 1846 points in the cross section. This data was exported as a data file into MATLAB and interpolated using a cubic interpolation onto a grid of 146 × 291 points equally spaced at intervals of 1 *μ*m. The velocity field on these grid points gave the undisturbed velocity of the fluid in the cross section with a resolution of 1 *μ*m. To verify that the slowly changing curvature of the channel had negligible effect on the flow, we also computed the velocity of the fluid at the cross section *C*_2_ (Fig. 1(a)) and found that the difference in the velocity of the fluid was negligibly small. The computed undisturbed velocity of the fluid as a function of the normalized distance from the inner wall at the mean particle focusing height from the bottom window is shown by the black solid curve in Fig. 3(a,b).

Figure 3(b) shows the velocities and positions (blue circles) of 20 different focused particles that converge to the lower focusing window. The velocities shown in the graph are normalized by the average velocity of the fluid, obtained by dividing the flow rate by the cross sectional area of the channel. The positions of the particles are normalized by the width of the channel. The positions of the centers of these particles measured from the channel’s inner wall range from 48 *μ*m to 106 *μ*m. The particles in this bottom focusing window clearly lead the flow, with the slip velocity being positive.

Figure 3(c) shows the velocity profile and positions (red circles) of 16 different focused particles that converge to the upper focusing window. The positions of these particles measured from the channel’s inner wall range from 68 *μ*m to 90 *μ*m. The particles in this upper focusing window lag the flow, with the slip velocity being negative. However the relative velocity in this case is a small fraction of the undisturbed flow velocity.

## Discussion of Results

**Figure 4:**
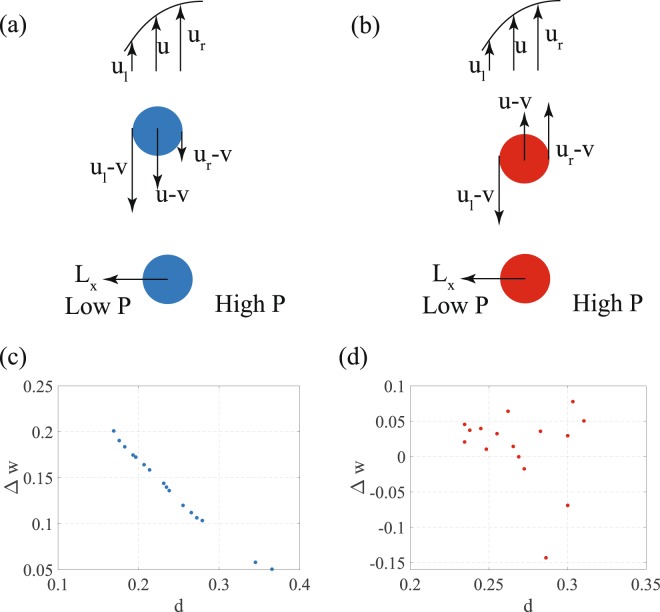
(**a** and **b**) A schematic of the axial velocity of the fluid and this velocity in a particle fixed frame of reference. The lower part of these figures showthe direction of the hydrodynamic lift force, *L*_*f*_ on the particles. (**c** and **d**) the relative axial velocity of the fluid, Δ*w*, diametrically opposite ends of particles in both the focusing windows.

Focused particles in the curved microchannel have an (axial) slip velocity. Particles which are injected at the inlet at an uncontrolled velocity converge to a narrow spatial region in the cross section of the channel and to a narrow range of slip velocities. In the lower focusing window a particle leads the flow. The velocity of the fluid relative to a particle in this window is *u* − *v* at the center of the particle, *u*_*l*_ − *v* at the left side of the particle (towards to the channel inner wall) and *u*_*r*_ − *v* at the right side (towards the channel center) of the particle. Due to the curvature of the velocity of the fluid shown in Fig. 4(a) the relative velocity of the fluid at the left side has a larger magnitude than at the right side, |*u*_*l*_ − *v*| > |*u*_*r*_ − *v|*. Figure 4(c) shows the difference in this relative velocities of the fluid with Δ*w* = |*u*_*l*_ − *v*| − |*u*_*r*_ − *v|*. The values of *u*_*l*_ and *u*_*r*_ are obtained from a numerical simulation in COMSOL. The higher relative velocity of the fluid on the left leads to a lower pressure on the left than on the right side of the particle, producing a hydrodynamic lift force, *L*_*f*_ pointing away from the channel centerline and towards the channel inner wall. The hydrodynamic lift force increases with increasing values of Δ*w*^9,10^. Figure 4(c) shows that Δ*w* decreases very quickly for particles that are farther from the inner wall implying that the farther away a particle is from the wall, the lower is the hydrodynamic lift force on it. In the lower focusing window, the transverse velocity of the fluid (in the Dean flow) is towards the inner wall and a particle experiences a transverse drag that acts to push it towards the inner wall. A third force that acts on the particles in the cross sectional plane of the channel is the wall induced lift force, which always acts to push particles away from the wall. The wall induced lift force is strong close to the wall and decreases rapidly away from the wall^11,24^. The net force due to the Dean drag force and the hydrodynamic lift is balanced by a wall induced lift that pushes the particles towards the center of the channel. The wall induced lift force required to maintain this balance is smaller as a particle is farther away since the hydrodynamic lift force too decreases. Such a trend is observed in Fig. 4(c) where the magnitude of Δ*w* decreases for particles farther away from the inner wall, which therefore experience a smaller inertial lift force.

In the focusing window located at about the channel mid height the particles lag the fluid such that the relative velocity of the fluid on the left side of the particles is in the opposite direction to that on the right side of the particle. However the difference, Δ*w* = |*u*_*l*_ − *v*| − |*u*_*r*_ − *v|*, is still positive for most of the particles as shown in Fig. 4(d). The result is that the pressure on the left side of the particle is lower, producing a hydrodynamic lift force on the particle towards the inner wall. This hydrodynamic lift force is balanced by the Dean drag force that acts to push the particles towards the center line and a weak wall repulsion that pushes the particles away from the inner wall.

The relative velocities of the particles in the focusing window located at the mid height are negative and very small in magnitude compared to those of the particles in the lower focusing window. The sign of these relative velocities could be sensitive to errors in the experimental procedure in the determination of the height of the particles from the channel bottom as well as the subsequent processing of the images to calculate particle velocities. If due to such errors the particles are found to lead the flow with a small relative velocity, the sign of Δ*w* would remain positive, i.e. relative velocity of the fluid on the side of the inner wall would still be smaller than the relative velocity of the fluid on the side of the outer wall. Therefore the direction of the hydrodynamic lift force would continue to point towards the inner wall as the analysis for the particles that lead the flow in the lower focusing window suggests.

There is a significant difference in the relative velocities of the particles in the focusing positions close to the bottom of the channel and the mid plane. The substantial relative velocity of the particle in the bottom focusing position is necessary to balance the vertical wall induced lift force. An analysis similar to the one illustrated in Fig. 4 can be performed for the vertical direction. The particles in the lower focusing positions lead the flow and the resultant Δ*w*_*y*_ the difference in the relative velocity across the height of a particle in this window is even larger. This produces a vertical lift force pointing towards the wall that balances the wall repulsion which itself is very high due to the closeness of the wall. The large shear gradient induced lift force is possible due to the large relative velocity of the particles.

The lift force coefficient due to the shear gradient that multiplies the relative velocity varies with the location in the cross section^25^ in a straight channel. It is also shown in^25^ that this lift coefficient is such that the lift force has a large vertical component close to the bottom and top of the channels and a negligible horizontal component. This is the opposite close to the mid plane. While the overall lift coefficient is smaller at the mid plane, the horizontal component is the dominant part of it. It has been reasonably assumed in the literature that the qualitative behavior of the lift coefficient is the same in spiral channels. Our data actually indicates this to be so. The smaller relative velocity and smaller Δ*w* is sufficient to balance the Stokes drag and the small wall induced repulsion at the mid plane height since the lift coefficient here has a mostly horizontal component. The larger relative velocity at the bottom focusing position is necessary since the lift coefficient has a dominant term in the vertical direction and negligible component in the horizontal direction. The large vertical lift force generated by the relative velocity balances the wall induced repulsion which is very strong close to the bottom surface.

We did not observe a third focusing window close to the top of the channel, symmetrically above the lower focusing window, where a sufficiently large number of particles could be observed. We attribute this to two possible reasons. The first is that our technique of finding particle slip velocity is not refined enough to consistently and clearly image particles close to the upper surface of the channel,when only a few particles migrate to such a position. A second possible reason is that the particles are not perfectly neutrally buoyant, with many of them having a specific weight that exceeds 1 by a very small amount. The few particles that are perfectly neutrally buoyant could be focused in the upper window. This agrees with our observation of finding only a few particles in an upper focusing window. For heavier particles, in a hypothetical upper focusing position, the weight of the particle, the wall repulsion and the vertical component of the Dean drag force point away (downward) from the top wall. To balance these through the hydrodynamic lift force requires a larger positive relative velocity of the particles. Such a large slip velocity would not be allowed due to the Stokes drag force in the axial direction.

Vertically symmetric focusing windows for particles are observed or have been suggested to exist in other work, see for instance^26–28^, where the experiments were conducted in a different regime of Reynolds-Dean number or channel geometry. For instance the experiments in^28^ used a channel with a high aspect rectangular cross section and small flow rates while^26^ used considerably higher Reynolds numbers (about 90–110). While it is understood through many experiments that channel Reynolds number, Dean number and channel cross section geometry effect particle focusing, our findings suggest another possible sensitivity to these parameters. At sufficiently high flow rates or very low flow rates (particle Reynolds number, *Re* = *Reλ*^2^ becomes negligible), the slip velocity could in fact be very small. When channel Reynolds number is very high a significant inertial lift force could exist without a slip velocity, due to the inertia of the fluid^29^ since Δ*w* is not zero even if *w* = 0. When the channel Reynolds number is very low, the lift force on the particle could be entirely dominated by the wall induced lift, leading to particle focusing positions close to the top and bottom channel walls. At the intermediate channel Reynolds number, investigated in this paper, the particle slip velocity plays a significant role. At the same time this makes a focusing window close to the upper wall less robust.

## Conclusion

The observations presented in this paper demonstrate the influence of the relative velocity of particles on their equilibrium positions in a curved channel. Neutrally buoyant particles are shown to lead or lag the flow at different equilibrium positions, unlike in a Poiseuille flow where particles lag the flow^11^. It is demonstrated that particle focusing in curved channels depends on achieving an equilibrium in the spatial positions as well as the relative velocities of particles. The requirement of having an attractor in both the position and relative velocity space has an important bearing to several applications of inertial microfluidics. It may well be possible to refine the combination of active and passive methods that could take advantage of the slip axial velocity to focus or sort particles. For example a DEP (dielectrophoresis) force could impart small slip velocities to particles to shift or create equilibrium positions that otherwise may not exist in the flow. Essentially the lift force that scales linearly with the slip velocity can be changed without changing the flow using such techniques. The combination of such active-passive methods that harness the slip velocity dependent focusing could allow focusing and sorting to be performed in regimes of Reynolds or Dean number that is currently not possible.
